# Commercial Tobacco Endgame Goals: Early Experiences From Six Countries

**DOI:** 10.1093/ntr/ntae069

**Published:** 2024-03-28

**Authors:** Janine Nip, Louise Thornley, Robert Schwartz, Rob Cunningham, Mervi Hara, Luke Clancy, David Evans, Fenton Howell, Sheila Duffy, Hans Gilljam, Richard Edwards

**Affiliations:** University of Otago, Department of Public Health, Newtown, New Zealand; University of Otago, Department of Public Health, Newtown, New Zealand; University of Toronto, Toronto, Ontario, Canada; Canadian Cancer Society, Ottawa, Ontario, Canada; ASH Finland, Helsinki, Finland; Tobacco Free Research Institute Ireland, Dublin, Ireland; Health Service Executive, National Social Inclusion Office, Dublin, Ireland; Department of Health, Dublin, Ireland; ASH Scotland, Edinburgh, Scotland; Department of Global Public Health, Karolinska Institute, Stockholm, Sweden; University of Otago, Department of Public Health, Newtown, New Zealand

## Abstract

**Introduction:**

Tobacco use is a major threat to health globally. A number of countries have adopted “endgame goals” to minimize smoking prevalence. The INSPIRED project aims to describe and compare the experiences of the first six countries to adopt an endgame goal.

**Aims and Methods:**

Data were collected on the initial experiences of endgame goals in Canada, Finland, Ireland, New Zealand (Aotearoa), Scotland, and Sweden up to 2018. Information was collated on the nature of the endgame goals, associated interventions and strategies, potential enablers and barriers, and perceived advantages and disadvantages.

**Results:**

The INSPIRED countries had relatively low smoking prevalences and moderate-to-strong smoke-free policies. Their endgame goals aimed for smoking prevalences of 5% or less. Target dates ranged from 2025 to 2035. Except for New Zealand (Aotearoa), all countries had an action plan to support their goal by 2018. However, none of the plans incorporated specific endgame measures. Lack of progress in reducing inequities was a key concern, despite the consideration of equity in all of the country’s goals and/or action plans. Experience with endgame goals was generally positive; however, participants thought additional interventions would be required to equitably meet their endgame goal.

**Conclusions:**

There was variation in the nature and approach to endgame goals. This suggests that countries should consider adopting endgame goals and strategies to suit their social, cultural, and political contexts. The experiences of the INSPIRED countries suggest that further and more significant interventions will be required for the timely and equitable achievement of endgame goals.

**Implications:**

By 2018, six countries (Canada, Finland, Ireland, New Zealand (Aotearoa), Scotland, and Sweden) had introduced government-endorsed “endgame goals,” to rapidly reduce smoking prevalence to very low levels by a specified date. The nature and implementation of endgame goals were variable. Early experiences with the goals were generally positive, but progress in reducing smoking prevalence was insufficient, particularly for priority groups. This finding suggests more significant interventions (“endgame interventions”) and measures to reduce inequities need to be implemented to achieve endgame goals. Variation in the nature and experience of endgame goals demonstrates the importance of designing endgame strategies that suit distinct social, cultural, and political contexts.

## Introduction

Tobacco use claims more than eight million lives per year and is a leading cause of mortality.^1^ Tobacco control interventions and strategies have been widely implemented, resulting in reductions in smoking prevalence in most countries.^2,3^

“Endgame thinking” urges action to achieve rapid and profound reductions in smoking prevalence.^4,5^ Endgame (or “smoke-free”) goals aim to reduce tobacco smoking (and in some cases, nicotine use) to a low level (eg, less than 5% prevalence) by a specified and reasonably imminent date. Finland became the first country to adopt a government-endorsed endgame goal in 2010. Endgame goals were subsequently introduced by the governments of New Zealand (Aotearoa) (2011), Scotland (2013), Ireland (2013), Sweden (2016), and Canada (2018).

Many commentators have argued that achieving profound and rapid reductions in the prevalence of tobacco use requires new and more significant approaches, sometimes termed “endgame interventions.”^5^ Endgame interventions that have been proposed include extremely large tobacco tax increases,^6^ mandated removal of nicotine from tobacco products,^7,8^ greatly reducing the retail availability of tobacco products,^9–11^ progressively increasing the minimum age of purchase or use,^12^ or introducing a “license to purchase” for people who smoke.^13^

There has been subsequent progress toward introducing endgame goals in other countries and regions. For example, Australia and the United States of America recently implemented targets of <5% smoking prevalence by 2030, the United Kingdom has set a target of being smoke-free by 2030, and Bangladesh recently introduced the goal of being tobacco-free by 2040.^5^

Documenting the early experiences of the initial adopters of endgame goals could provide valuable information for other countries and regions that are considering implementing endgame goals or have recently introduced them.

The INSPIRED project (International Network to Share Insights on Tobacco Endgames) aims to describe, compare, and contrast the experience of the first six countries to adopt an endgame goal. This includes exploring the nature and context of each endgame goal, associated interventions and strategies, potential enablers and barriers, and perceived advantages and disadvantages. This article presents findings of the early experiences of the endgame goals in the INSPIRED countries.

## Methods

This study included countries that, by 2018, had a government-endorsed quantifiable national endgame goal, such as the reduction of smoked tobacco product use or supply to a low level, with a target date before 2040. The goal could be defined as an adult smoking prevalence of 5% or less, smoking uptake of less than 2% in adolescents, and/or no or minimal supply and sale of smoked tobacco products.

In December 2017, we approached tobacco control experts in Finland, Ireland, New Zealand, Scotland, and Sweden about taking part in the study. We invited Canadian colleagues in February 2018. Additional key people were identified by in-country experts to contribute to data collection, such as tobacco control practitioners, researchers, advocates, and policy-makers. This resulted in a total of 38 participants from the six countries (see Acknowledgments).

Data collection templates were filled in by participants from each country. The templates included questions relating to smoking prevalence, definition of the endgame goal, and the presence or absence of a tobacco endgame action plan or strategy (please see Appendix Table 1 for a copy of the template). The templates also recorded key tobacco control interventions that were in place or planned, including interventions listed in the World Health Organization (WHO) MPOWER package.^2,14^ Core interventions outlined in the package include smoke-free policies, smoking cessation support, public education, bans on advertising and promotion, and increasing tobacco excise taxes.^2,3^ Participants were asked to identify advantages and disadvantages of having an endgame goal, whether they thought their country was likely to achieve its endgame goal, and the main enablers and barriers to reaching their endgame goal.

Templates were returned by February 2018, with the exception of Canada, which provided information in July 2018. Further information was collected where required via queries to the in-country collaborators. The University of Otago study team also incorporated additional information from an analysis of key documents and information available online. Ethical approval was obtained from the University of Otago (reference number: D22/331).

Please see the full “2018 INSPIRED Report” at https://aspireaotearoa.org.nz/our-research/current-research/inspired for further detail, including country-specific data.

## Results

### Smoking Prevalence

Adult smoking prevalence by 2018 for each of the INSPIRED countries is demonstrated in Table 1. A WHO analysis reported that the mean absolute decline in smoking prevalence was between 0.58% and 0.84% per year in the six countries between 2005 and 2015.^15^ The rate of reduction for each INSPIRED country was higher than the mean rate of reduction across all countries (mean 0.41% per year) and all high-income countries (mean 0.55% per year) included in the WHO analysis.

**Table 1.** Country-Specific Smoking Prevalence Trends and Endgame Goals

Country	Annual rate of decline in current smoking prevalence among adults 2005–2015^*^	Daily adult tobacco smoking prevalence in 2018^**^	Endgame goal				Annual decline in daily smoking (%) required to achieve 5% prevalence by the endgame target year
			Year introduced	Target(s)	Interim target(s)	Target date	
*Canada* ^16^	−0.78%	11%^17^ [16% current^†^]^17^	2018	<5% adult current tobacco use prevalence	Nil	2035	0.34% per year for 17 years^††^
*Finland* ^18^	−0.58%	12%^19,20^	2010^#^	<5% daily use of all tobacco and nicotine products, including nicotine e-cigarettes^#^	Nil	2030^#^	0.58% per year for 12 years
*Ireland* ^21^	−0.84%	17%^22^	2013	<5% adult current smoking prevalence	Nil	2025	1.71% per year for 7 years
*New Zealand* ^23^	−0.79%	13% (note data are from 2017/2018)^24^	2011	≤5% adult daily smoking prevalence Minimal availability of tobacco products	Daily smoking prevalence in 2018: ^##^ ≤10% for total population; ≤19% for Māori; ≤12% for Pacific peoples	2025	1.14% per year for 7 years
*Scotland* ^25,26^	−0.69% (UK)	19% current ^†††^^,27^	2013	≤5% adult current smoking prevalence Tobacco-free generation, where children born from 2013 would form a tobacco-free generation	Smoking prevalence in: ^^^ 2021: <20% for the two most deprived quintiles combined; 2022: <3% regular smokers among children aged 13–15 years; 2023: <20% smoking prevalence among 20–24 year olds	2034	0.88% per year for 16 years
*Sweden* ^28^	−0.76%	7%^29^	2016	<5% adult daily smoking prevalence	Nil	2025	0.29% per year for 7 years

^*^Predictions from World Health Organization estimates extrapolating from available survey data using standardized methodologies.^15^ Of note these figures are in keeping with reported reductions in smoking rates over time in participating countries, please see the full “2018 INSPIRED Report” at https://aspireaotearoa.org.nz/our-research/current-research/inspired for detail. Data on annual decline are absolute data. Please see Appendix Table 2 for data on the daily adult smoking prevalence in the year each endgame goal was introduced.

^**^As reported in population-based surveys, figures may not be fully comparable because of differences in sampling approaches, and age-ranges of participants included in the surveys.

^†^Current smoking estimates are provided for Canada because in-country informants noted some doubts about validity of daily smoking prevalence estimates. The 16% estimate is preferred by Canada participants in this project, as it is considered more accurate than the daily smoking estimate. Similar figures were reported for over 15 year olds in the biennial Canada Tobacco, Alcohol and Drugs survey in 2017.^30^

^††^Value is 0.64% per year for current smoking.

^†††^Current smoking estimates are provided for Scotland as no daily smoking prevalence data available.

^#^The initial endgame goal was <2% prevalence of smoking and the target date was 2040. However, in 2016 the goal was updated to include all nicotine products, goal was set to less than 5%, and the target date was brought forward to 2030.

^##^These targets were not met.

^^^These targets were set in 2018. More detailed interim targets were also set in 2013.

Inequities in smoking prevalence by ethnicity were noted in a number of countries. For example, in Canada, smoking prevalence was two to five times higher in Indigenous people than the rest of the population. In New Zealand, smoking prevalence in Māori was over twice the smoking prevalence in people who identified as European/Other.^24^ In Ireland, the Traveler Community had a smoking rate of 53% in 2010, compared to 23% in the overall population.^21,31^ The Traveler community in the Equal Status Acts is defined as “the community of people who are commonly called Travelers and who are identified (both by themselves and others) as people with a shared history, culture and traditions, including historically, a nomadic way of life on the island of Ireland.” Further information can be found at https://www.ihrec.ie/download/pdf/traveller_ethnicity.pdf.

Inequities by socioeconomic status were also noted, with higher smoking prevalence associated with lower income (Canada^32^ and Sweden^29^), lower education level (Finland^19^ and Sweden^29^), and living in a more deprived area (Ireland,^22^ New Zealand,^24^ and Scotland^27^). For further details on inequities by socioeconomic status and proposed actions to address them, please see Appendix (Summary 1 and Table 3, respectively).

### Endgame Goals

As outlined in Table 1, every country had an endgame goal to reduce adult smoking prevalence to <5% or ≤5%. However, some goals included additional components; Finland included the elimination of all nonmedical nicotine products; Canada included use of all tobacco products; and New Zealand included a goal of reducing availability of tobacco to minimal levels. Target dates ranged from 2025 to 2035. As detailed in Table 1, New Zealand and Scotland were the only countries to specify interim smoking prevalence goals for priority population groups. In New Zealand, specific interim targets were set for Māori, and Pacific peoples. In Scotland, they were set by deprivation quintile and age group.

### Action Plans/Strategies to Achieve Endgame Goals

In 2018, each of the INSPIRED countries had a government-endorsed action plan or strategy in place to achieve their endgame goal, except for New Zealand. As detailed in Appendix Table 2, all of the action plans/strategies had a clear focus on reducing smoking rates in priority population groups.

#### Tobacco Control Interventions in Place in 2018

Participating countries had partially or fully implemented most measures from the WHO MPOWER list of tobacco control interventions.^2,14^ Every country performed well (highest or second highest category) for monitoring smoking prevalence, having health warnings on cigarette packets, providing smoking cessation support, advertising bans, and tobacco taxation.

Performance was more variable with regards to smoke-free policies and mass media campaigns. While Canada, Ireland, New Zealand, and Scotland were in the highest category for the implementation of smoke-free policies, Sweden and Finland were in the lowest MPOWER category (“complete absence of a ban, or up to two public places completely smoke-free”).^2^ Mass media campaigns ranged from no national campaigns in Canada to national campaigns that aired on television and/or radio in Ireland, New Zealand, and Scotland.

All six countries, except Sweden, had implemented additional interventions beyond MPOWER measures. These included restrictions on retailers and retailer registration/licensing (Canada, Finland, Ireland, and Scotland), prohibition of certain tobacco flavors (Canada, Finland, and Scotland), regular tobacco tax increases (Finland, Ireland, and New Zealand), enhanced smoke-free laws such as smoke-free cars legislation (Finland, Ireland, Scotland, and some provinces in Canada), bans on point-of-sale displays and advertising (Finland, Ireland, New Zealand, Canada, and Scotland), and plain packaging regulations (Ireland and New Zealand).

#### Approach to Alternative Nicotine Products

In 2018, the sale of e-cigarettes was legal in all INSPIRED countries, with varying restrictions on use and availability. The prevalence of e-cigarette use was lower than the daily adult tobacco smoking prevalence for each country. For further details on e-cigarette use prevalence, please see Appendix (Summary 2).

The approach to the use of alternative nicotine and/or tobacco products was different in each country. Finland’s roadmap for achieving their endgame goal explicitly rejected the use of alternative tobacco products as a means to reduce harm from smoking. In keeping with this, Finland had the only endgame goal that incorporated ending the use of all tobacco and nicotine products, including e-cigarettes (Table 1). Snus sale is illegal in Finland; however, people can privately import restricted amounts for their personal use. In 2018, daily snus use was 4.9% among men and 0.2% for women.^20^ In 2017, 8% of adolescents (14–18 years) used snus daily or occasionally.

In contrast, Scotland and Canada considered that alternative cessation products may have roles as potential smoking cessation aids in their action plans/strategies. In 2018, Ireland was in the process of developing formal government positions in this area. In addition, Canada has recently moved to introduce a regulatory framework for nicotine-containing e-cigarettes and other alternative products.

In New Zealand, nicotine-containing e-cigarettes were initially banned from sale. However, in November 2018, new proposals were agreed upon by the New Zealand Cabinet for a regulatory framework for nicotine-containing e-cigarettes, which would allow widespread retail availability with regulation via restrictions on age of purchase, quality standards, place of use, and advertising.

In Sweden, there was a long-standing and widespread availability and use of snus, but there was no official government position on snus and it was excluded from their tobacco control action plan.

### Proposals and Plans for New Actions

In 2018, a variety of new tobacco control interventions were planned in each country. These were mostly incremental in nature and no country was actively considering the introduction of more specific “endgame interventions.” Please see Appendix Table 3 for further detail.

### Perceived Advantages and Disadvantages of Having an Endgame Goal

Participants in the INSPIRED countries identified a number of advantages and some potential disadvantages of having a tobacco endgame goal, as outlined in Table 2.

**Table 2.** Perceived Advantages and Disadvantages of Adopting an Endgame Goal

Key advantages	Key disadvantages
• Enhancing clarity of purpose for the tobacco control sector. • Increasing political priority and societal and public support for tobacco control actions. • Enabling the introduction of tobacco control measures and increased resource allocations for tobacco control activities. • Providing tobacco control stakeholders with an anchor for generating new ideas and advocacy for stronger tobacco control measures, resources, and/or calls for further research.	• Focus on setting or debating endgame goals could distract from achieving implementation of key tobacco control interventions. • Potential negative impacts of failure to achieve endgame goals, such as reduced motivation and increased pessimism about tobacco control efforts. • It could be difficult to manage expectations and ensure the public understand that greater effort is needed to achieve the endgame goal. • A long-term vision may be seen as abstract and hence be easily overlooked.

### Perception As to Whether Endgame Goals Would Be Met

Participants from Sweden reported that the likelihood of achieving their goal was high. They noted that Sweden had a comparatively low smoking prevalence and that their goal had already been met for high socioeconomic groups. However, participants felt that strong political commitment and additional measures would likely be required to reach the endgame goal for all population groups.

Participants from the other INSPIRED countries were less convinced that endgame goals would be achieved overall and for all population groups. They all reported that new interventions would be needed for their country to meet their endgame goal by its target date.

Participants from Finland referred to a 2017–2018 qualitative study in which key stakeholders reported that they believed Finland was on track to reaching their endgame goal.^33^ However, the authors recommended that the following improvements would be required: improved coordination between nongovernmental organizations (NGOs) and the healthcare system, agreement about the place of mass media campaigns, and strengthening of smoking cessation services.

Participants from Canada and Ireland reported that their endgame goal may be achievable for the population overall, but not for all population subgroups. They highlighted that new actions must be introduced if their endgame goals are to be met. In Canada, SimSmoke modeling estimated smoking prevalence would be 8.5% by their target endgame date (2035) if plain packaging, free cessation services, decreased tobacco availability, and increased tobacco taxation were implemented.^34^ In Ireland, SimSmoke modeling found that smoking prevalence could be reduced to 12.4% by their endgame target date (2025) if stronger MPOWER-compliant policies were implemented.^35^ The study authors also suggested that new and innovative policies that go beyond the conventional MPOWER measures were required to reach the goal.

In Scotland and New Zealand, prespecified interim endgame targets for smoking prevalence had not been met. Participants from New Zealand believed they were unlikely to reach their endgame goal through current interventions.^36–40^ There was particular concern that targets would not be met for Māori, with modeling predicting that without additional interventions, Māori smoking prevalence would not reach <5% until at least 2060.^36^ However, modeling of the impact of interventions like substantial tax increases, reductions in retail supply, and a tobacco-free generation strategy suggested the Smoke-free 2025 endgame goal could be achieved for the whole population, and that there would be large reductions in smoking prevalence among Māori. In Scotland, participants reported that the rate of decline in smoking prevalence was decreasing. However, it was also noted that there had been a steep drop in smoking among 13–15 year olds, and that if smoking initiation continues to decline sharply there is a “good chance” that Scotland can get back on track to achieving their 2034 endgame goal.

### Perceived Enablers, Barriers, and Critical Readiness Factors for Introducing and Achieving Endgame Goals

As summarized in Table 3, participants identified a number of critical readiness factors for initiating an endgame goal, as well as enablers and barriers to achieving their countries’ endgame goal.

**Table 3.** Perceived Enablers, Barriers, and Critical Readiness Factors for Introducing and Achieving Endgame Goals With Examples From INSPIRED Countries

Perceived enablers	Perceived barriers
• Strong political support for the endgame goal and actions,^*^ including political champions^*^ • Government strategy, interim targets, review mechanisms and research. • **Focus on reducing disparities in smoking prevalence, including Indigenous communities, with importance placed onIndigenous leadership**.^*^ • **Implementing robust tobacco control measures**.^*^ • Strong cross-sectoral collaborative structures^*^ to facilitate and monitor progress towards an endgame goal. • **Public support for endgame goals**.^*^	• Insufficient priority for the endgame goal. • Lack of funding, capacity, and resources for tobacco control. • Insufficient progress on reducing disparities in smoking. • Lack of implementation of leading-edge, innovative interventions, such as “endgame measures.” • Tobacco industry influence and actions. • Lack of unity in the tobacco control sector.

NGO = nongovernmental organization.

^*^Factors in bold, along with a relatively low and/or rapidly declining prevalence of smoking, were identified by participants as critical readiness factors for a country to implement an endgame goal. For full detail see the 2018 INSPIRED Report at https://aspireaotearoa.org.nz/our-research/current-research/inspired.

#### Perceived Enablers Included:

1. *Strong political support for the endgame goal and actions*: This included sustained political commitment, backing from civil society and the public to help maintain political commitment, and identifying and supporting influential political champions. Most of the INSPIRED countries had one or more political champion(s) who had helped drive the idea of adopting an endgame goal for tobacco smoking.
2. *Government strategy, interim targets, review mechanisms, and research:* Scotland, Ireland, and Finland reported especially strong monitoring and review mechanisms. Scotland and Ireland, for example, annually monitored progress with their endgame goals. Finland regularly reviewed and updated their strategy, incorporating monitoring of prevalence and intervention implementation. By contrast, New Zealand and Sweden had no regular formal review processes in place. Tobacco control researchers and research program were present in all INSPIRED countries.
3. *Focus on reducing disparities in smoking prevalence, including Indigenous communities, with importance placed on Indigenous leadership:* Each of the INSPIRED countries included a stated focus on reducing smoking rates in priority population groups in their endgame goal and/or government action plans/strategies.
4. *Implementing robust tobacco control measures:* Participants agreed that endgame tobacco control measures are likely to be needed to achieve endgame goals.
5. *Strong cross-sectoral collaborative structures to facilitate and monitor progress towards an endgame goal:* National cross-sector collaborations and formal partnerships between NGOs and government agencies were present in Canada, Finland, Ireland, Scotland, and Sweden. Some were led by government; others were led by NGOs. New Zealand’s cross-sectoral structures were less well developed. Participants noted that the introduction of a government-endorsed endgame goal was generally preceded by debate and discussion about endgame goals and ideas within the tobacco control and/or public health sectors in each country, and often the adoption by these sectors of an endgame goal preceded the official government goal.
6. *Public support for endgame goals:* All of the INSPIRED countries had evidence of strong public support for the endgame goal and/or key tobacco “endgame interventions.”^41–46^

#### Perceived Barriers Included:

1. *Insufficient priority for the endgame goal:* Participants in Canada, Scotland, Sweden, and New Zealand reported that, despite stated government support, the level of government commitment to their endgame goal was in practice often variable and less than for other competing priorities—especially at times of limited resources. Endgame goals had to compete with other priorities in politics, legislation, and/or resource allocation. Participants in Finland and Scotland both mentioned the challenge of sustaining the priority of endgame work over a long timeframe. In New Zealand, a key barrier was the absence of a government-endorsed action plan or strategy by 2018, despite adoption of their endgame goal in 2011. In contrast, Ireland was a recognized leader in European tobacco control, including a crucial role in advocating to secure the European Union Tobacco Products Directive and political commitment to tobacco control was demonstrated by the early introduction of a tobacco control strategy. In Finland, there was also broad political support for tobacco and nicotine-free Finland, and tobacco control legislation has been consistently developed regardless of fierce opposition from the tobacco industry.
2. *Lack of funding, capacity, and resources for tobacco control:* Examples provided by participants included: a lack of government capacity to “join up” strategies and drive progress (Scotland), insufficient investment in smoking cessation support (Finland), and a lack of nationwide communication campaigns (Finland). Participants in Canada reported insufficient government budgetary investment in tobacco control, including for cessation and other initiatives. Swedish, New Zealand, and Canadian participants also reported a lack of sufficient funding specifically for tobacco control actions and NGOs.
3. *Insufficient progress on reducing disparities in smoking:* A lack of success in tackling socioeconomic and/or ethnic disparities in smoking prevalence was seen as a major constraint on progress towards endgame goals. Persisting disparities in smoking were apparent in all of the INSPIRED countries, including Indigenous groups in New Zealand, Canada, Ireland, and Sweden.
4. *Lack of implementation of leading-edge, innovative interventions, such as “endgame interventions”:* A broad mix of additional, cutting-edge interventions had been proposed by the tobacco control sectors in Canada and New Zealand; however, in 2018 these were not yet being considered by government. Ireland was the only country to introduce legal or fiscal measures to increase tobacco industry accountability for the harms caused by their products. The Irish Strategic Investment Fund had completed the sale of its remaining investments in tobacco shares and a Bill was before the Oireachtas (Parliament) to prohibit the investment of public monies directly or indirectly in equity or debt securities issued by tobacco companies. Finland had proposed banning public investment in the tobacco industry, and Canada’s strategy proposed future measures to increase industry accountability for tobacco-caused harm. In Scotland, there was advocacy and discussion on a tobacco industry levy at the UK and/or Scottish level.
5. *Tobacco industry influence and actions:* In all six countries, tobacco industry opposition and interference were identified as a key barrier to progress. Participants cited examples of tobacco industry lobbying against tobacco control measures, as well as lobbying from industry-funded organizations, such as retailer associations, “think-tanks,” or research consultancies. Participants noted that tobacco industry influence, lobbying, and legal challenges had impeded progress with some interventions, such as the introduction of plain packaging and point-of-sale restrictions in Scotland and Ireland.
6. *Lack of unity in the tobacco control sector:* Participants from New Zealand and Scotland stated that disagreement among tobacco control experts and key stakeholders over alternative tobacco products and harm reduction was potentially affecting endgame progress. For example, several New Zealand tobacco control leaders have said the debate over e-cigarettes (and other alternative nicotine-delivery products) had the potential to distract, fragment, and weaken the tobacco control sector.

## Discussion

### Key Findings and Implications

The INSPIRED study describes the experiences of the first six countries to introduce endgame goals to facilitate more rapid reductions in smoking prevalence. With more countries introducing endgame goals, there are possible insights from the experiences of early adopters, all of whom have set targets to reduce smoking prevalence to 5% or less.

A key insight is that endgame goals can have positive impacts prior to the goal being achieved. For example, early adopters have significantly accelerated declines in smoking prevalence compared to countries that have not set goals.

Another key insight is that potential key drivers for the achievement of endgame goals were the accompanying strategies, action plans, and review mechanisms, which were heterogeneous across the six INSPIRED countries. With limited evidence about the best approaches to achieve endgame goals, this seems appropriate, and it is unlikely that a “one-size-fits-all” solution will emerge. For example, there was diversity among early adopters toward regulatory frameworks and policies implemented for e-cigarettes and other alternative nicotine products. The heterogeneity of approaches underlines the need for comprehensive evaluation of endgame goals and strategies to facilitate subsequent decision-making. There is also scope to undertake research prior to implementation by modeling the potential impacts of different measures to inform strategies and approaches.

Each of the INSPIRED countries had a strong focus on the importance of reducing inequities and achieving endgame goals for all population groups, in addition to minimizing overall smoking prevalence. However, by 2018, there was limited progress in reducing socioeconomic and ethnicity-based disparities in smoking prevalence in each country. These findings suggest practical steps to reduce inequities should be included in endgame strategies and action plans at the outset. These could include identification of at-risk population groups with high smoking prevalence and/or disparities in smoking-related harms, engagement with at-risk groups to identify their priorities and how they can be addressed, implementation of interventions which have been demonstrated to be proequity, and evaluating progress in at-risk groups (including assessment of smoking prevalence and evaluation of any unanticipated impacts of tobacco control interventions).^5,47–49^

The study identified enablers and barriers to endgame goals. These could be considered by countries contemplating introducing endgame goals and those that are currently progressing them. For example, cross-party support was identified as an important enabler. Its relevance was demonstrated recently in New Zealand, where a change in government led to a complete policy reversal through the repeal of the Smoke-free Environments and Regulated Products (Smoked Tobacco) Amendment Act introduced by the previous government in early 2023.^50^ As a result, three groundbreaking endgame interventions (a substantial reduction in the number of places able to sell smoked tobacco products from July 2024, mandated denicotinization of cigarettes from April 2025, and making it illegal to sell smoked tobacco products to people born on or after January 1, 2009) will not be implemented. This illustrates why it is important to try and achieve cross-party support for endgame strategies and measures wherever possible to ensure their full implementation.

A key finding was that, at the time of the study, it was debatable as to whether any of the countries had implemented or were even considering “endgame interventions,” yet all participants stated that further interventions were required for their endgame goals to be equitably reached by their target dates. Participants from Sweden, for example, highlighted that whilst their country appeared “on track” to achieve their endgame goal, further interventions would be required to reach their goal for all population groups. This highlights the need for countries to consider the implementation of a comprehensive set of tobacco control measures, probably including “endgame interventions” in order to achieve endgame goals equitably.^5–13^

### Limitations of This Study

The inclusion of only high-income Western countries may limit the generalizability of our findings. For example, our study results may not be as applicable to the implementation of tobacco endgame goals or strategies in low- or middle-income countries. Additionally, we provide information only on national-level goals—experience from similar local, regional (eg, the Tobacco Free Pacific 2025 goal^51^ and Europe’s Beating Cancer Plan^52^) or even global endgame goals (eg, the proposed 2040 tobacco-free goal^53^) may be different.

The study focuses on early adopters of endgame goals up to 2018. Further developments since are not considered in the results. However, critically assessing early experiences in the INSPIRED countries has generated valuable insights that may assist other policy-makers, tobacco control practitioners, and advocates about how best to introduce and progress endgame goals.

We acknowledge that the information collected in this study relating to the experience of endgame goals in each country reflects participants’ opinions. Other tobacco control experts in the INSPIRED countries may hold different views.

## Conclusions

Endgame thinking and the introduction of endgame goals represent a paradigm shift and a significant development in global tobacco policy. Endgame thinking has the potential to accelerate progress toward tobacco-free societies. Countries and regions considering adopting endgame goals and measures may learn important insights from the early experiences of the countries which first introduced endgame goals. The findings from these early adopters are largely positive and suggest endgame goals are supported by the public,^41–46^ can help facilitate the introduction of robust tobacco control measures and strategies, and may accelerate reductions in smoking prevalence.

However, it is early days in the study of tobacco endgames. Questions remain about the future progress and outcomes of country-level endgame goals and the merit of different strategies that could be used to achieve them, including the potential contribution of alternative nicotine products. This should be addressed through ongoing research and collaboration to monitor experiences and evaluate the impacts of endgame goals, strategies, and interventions.

## Supplementary Material

Supplementary material is available at *Nicotine and Tobacco Research* online.

ntae069_suppl_Supplementary_Materials

## Data Availability

Further detail is available in the full “2018 INSPIRED Report” at https://aspireaotearoa.org.nz/our-research/current-research/inspired. The authors will also be available to respond to specific queries on request.
