# Correction to: Commercial Tobacco Endgame Goals: Early Experiences From Six Countries

**DOI:** 10.1093/ntr/ntae132

**Published:** 2024-06-04

This is a correction to: Janine Nip, Louise Thornley, Robert Schwartz, Rob Cunningham, Mervi Hara, Luke Clancy, David Evans, Fenton Howell, Sheila Duffy, Hans Gilljam, Richard Edwards, the INSPIRED collaboration, Commercial Tobacco Endgame Goals: Early Experiences From Six Countries, *Nicotine & Tobacco Research*, 2024;, ntae069, https://doi.org/10.1093/ntr/ntae069.

The order of citations in the third paragraph has been adjusted for accuracy.

