# Supplementary material for: Commercial Tobacco Endgame Goals: Early Experiences From Six Countries
Source: Nicotine Tob Res. 2024 Mar 28;26(10):1322–30. doi: 10.1093/ntr/ntae069 (PMC11417120; doi:10.1093/ntr/ntae069)
Supplement: ntae069_suppl_Supplementary_Materials [file ntae069_suppl_supplementary_materials.docx]

**APPENDIX: Commercial Tobacco Endgame Goals: Early experiences from six countries**

Janine Nip PhD^1^, Louise Thornley Master of Arts^2^, Robert Schwartz PhD ^3^, Rob Cunningham LLB, MBA ^4^, Mervi Hara M.Sc. (Pol.) ^5^, Luke Clancy MD^6^, David Evans PhD^7^, Fenton Howell FFPHMI ^8^, Sheila Duffy MA Hons (Cantab)^9^, Hans Gilljam MD^10^, Richard Edwards MD^1^, on behalf of the INSPIRED collaboration.

1. University of Otago, Department of Public Health, PO Box 7343, Newtown, Wellington 6242, Aotearoa New Zealand
2. Previously working with University of Otago, Department of Public Health, PO Box 7343, Newtown, Wellington 6242, Aotearoa New Zealand
3. University of Toronto, 27 King’s College Cir, Toronto, Ontario, Canada
4. Canadian Cancer Society, 116 Albert Street, Ottawa, Ontario, Canada
5. ASH Finland, Helsinki, Valimotie 17-19, Helsinki, Finland
6. Tobacco Free Research Institute Ireland, TUDublin, Dublin 2, D02 HW71, Ireland
7. Health Service Executive, National Social Inclusion Office, Mill Lane, Palmerstown, Dublin 20, D20 KH63, Ireland
8. Department of Health, Block 1, Miesian Plaza, 50 – 58 Lower Baggot Street, Dublin 2, D02 XW14, Ireland
9. ASH Scotland, 8 Frederick St, Edinburgh, Scotland
10. Dpt. of Global Public Health, Karolinska Institute, Solnavägen 1, 171 77 Solna, Stockholm, Sweden

**Corresponding author:**

Dr Janine Nip

University of Otago, Wellington

PO Box 7343, Newtown, Wellington 6242, New Zealand

Janine.nip@otago.ac.nz

+64 3 479 7000

**APPENDIX TABLE 1: Template for data collection**

# Template – Tobacco endgame goal status

## Part 1: Tobacco endgame context and learning

| **1.1 COUNTRY CONTEXT** | |
| --- | --- |
| **Type of government** |  |
| **Population (eg, ethnicity breakdown)** |  |
| **Origins of endgame goal (including who came up with the idea)** |  |
| **Particular features of tobacco product use (eg, widespread Snus use in Sweden)** |  |
| **Any other contextual information?** |  |
| **1.2 SMOKING PREVALENCE AND DISPARITIES** | |
| **Brief summary of adult smoking prevalence, including by gender** |  |
| **Brief summary of young adult smoking prevalence (eg, 18-24 yrs), including by gender** |  |
| **Brief summary of adolescent smoking prevalence (eg, 15-17 yrs)** |  |
| **Evidence of any disparities in tobacco use (eg, by ethnicity, socio-economic status)** |  |
| **Summary of data on use of e-cigarettes and other products such as waterpipe tobacco, smokeless tobacco (eg, levels of use by adults and youth, concurrent use with tobacco smoking)** |  |
| **Evidence that goal will be met (or not) on current trends** |  |
| **Do you have any other comments on prevalence or disparities?** |  |
| **1.3 ENDGAME GOAL** | |
| **Date adopted** |  |
| **Title and wording of the goal. Is there a percentage target specified for reducing smoking prevalence?** |  |
| **Specific outcome sought. Does the goal focus on minimising harm or abstinence from nicotine?** |  |
| **Timeline (eg, interim and final targets)** |  |
| **Author/publisher of goal document** |  |
| **Any other information about the goal?** |  |
| **1.4 NATIONAL STRATEGY OR PLAN** | |
| **Has a national strategy/plan to achieve the endgame goal been produced? Is this a government plan or another plan (eg, by civil society/NGOs, advocates or academics)?** |  |
| **If a plan exists, is it a dedicated plan or part of another plan (eg, on NCDs or combined with alcohol and other drugs)** |  |
| **Briefly list the key actions in the current plan** |  |
| **To what extent is the plan being implemented?** |  |
| **How successful are the actions likely to be in progressing towards the goal? (in your view)** |  |
| **Any other comments on the plan?** |  |
| **1.5 COMMITMENT TO REDUCING SOCIAL AND ETHNIC DISPARITIES** | |
| **Is there specific reference to equity issues in the goal (and/or plan)?** |  |
| What level of priority is given to reducing disparities in the goal and/or plan? Does the plan include any targeted interventions? (briefly describe) |  |
| **Any other comments on reducing disparities?** |  |
| **1.6 CURRENT AND PLANNED INTERVENTIONS** | |
| **Brief summary of current interventions (summary only – further detail is in Part 2 below)**  **Interventions may be by government, civil society/NGOs, community, etc.S** |  |
| **Interventions planned in the next five years** |  |
| **1.7 POLITICAL SUPPORT AND RESOURCING** | |
| **Evidence of senior-level political support** |  |
| **Evidence of cross-party support** |  |
| **Evidence of commitment of resources – specifically to the endgame goal and to tobacco control overall** |  |
| **Degree of success of advocacy efforts in encouraging government to increase resourcing for the tobacco endgame goal** |  |
| **Evidence of cross-department working (eg, are some interventions and leadership coming from outside the Health Dept or Ministry?)** |  |
| **Evidence of infrastructure to implement and monitor progress (eg, national unit, evaluation framework and resources, government funded national monitoring survey/s)** |  |
| **Other evidence of political commitment** |  |
| **1.8 CIVIL SOCIETY SUPPORT** | |
| **Evidence of civil society support at a national level, eg, national advocacy or civil society coalition or advisory group** |  |
| **Evidence of one or more civil society organisations dedicated to achieving the goal** |  |
| **Evidence of civil society strategy for achieving the goal, eg, have strategy documents been produced?** |  |
| **Other evidence of civil society support** |  |
| **1.9 PUBLIC SUPPORT** | |
| **Evidence of public and smoker support for the endgame goal (please specify)** |  |
| **Evidence of public and smoker support for key interventions (brief summary/examples)** |  |
| **Any other information on public support?** |  |
| **1.10 LEARNING FROM COUNTRY EXPERIENCES** | |
| **What has worked well? What are the main facilitators to progress?** |  |
| **What has not worked? What are the main barriers to progress?** |  |
| **What advice would you give to other countries about endgame goals?** |  |
| **Do you have any other comments to make?** |  |

## Part Two: Checklist on current and planned interventions

**Intervention status = extent of implementation – please use this scale for responses under ‘Intervention status’ in the table below:**

1- fully implemented

2- partially implemented (give detail in notes)

3- not implemented but planned (give timeline in notes)

4- not implemented but under discussion

5- not implemented and not on agenda currently

| **Policy or intervention** | **Intervention status**   - **to what extent is it implemented?** | **Notes (include year of last major change and any planned new policies or interventions)** |
| --- | --- | --- |
| **Overall Tobacco Control Scale ranking (for European countries)** |  |  |
| **2.1 Tax and price** | | |
| Recent (within last 2 years) or planned (within next 2 years) above inflation increases in tobacco tax |  |  |
| Tobacco tax as % of retail price |  |  |
| For European countries, score for “price of cigarettes” indicator (out of 30 points)- and estimate for NZ |  |  |
| **2.2 Policies restricting where people can smoke** | | |
| National smokefree indoor workplace and public places policy/legislation |  |  |
| National smokefree outdoor (including cars) workplace and public places policy/legislation |  |  |
| For European countries, score for “smokefree work and other public places” indicator (out of 22 points) |  |  |
| **2.3 Public information campaigns** | | |
| Is a national tobacco control public information / mass media campaign in place? |  |  |
| What is the per capita spend on tobacco control campaigns/communications?  If known: of the total spend, how much is allocated to cessation, social marketing and information-only |  |  |
| For European countries, score for “spending on public information campaigns” indicator (out of 15 points) |  |  |
| **2.4 Advertising and marketing restrictions** | | |
| Is there a comprehensive ban on advertising, marketing and sponsorship of tobacco products? |  |  |
| Ban on advertising and display of tobacco products in retail settings (point-of-sale) |  |  |
| For European countries, score for “comprehensive bans on advertising and promotion” indicator (out of 13 points) |  |  |
| **2.5 Tobacco product packaging** | | |
| Plain packaging of tobacco products |  |  |
| Tobacco pack warnings size |  |  |
| Pictorial health warnings |  |  |
| For European countries, score for “large, direct health warning labels” indicator (out of 10 points) |  |  |
| **2.6 Smoking cessation support** | | |
| Dedicated cessation support network |  |  |
| Reimbursement of cessation support medications |  |  |
| Quitline |  |  |
| Systems to support cessation support in primary health care, eg, brief advice in primary care, recording of smoking status in medical notes |  |  |
| For European countries, score for “treatment to help smokers stop” indicator (out of 10 points) |  |  |
| **2.7 Restrictions on use by and sales to minors** | | |
| Age restrictions on sale/use |  |  |
| **2.8 E-cigarettes/e-liquids etc** | | |
| Availability of nicotine-containing e-cigarettes and e-liquids |  |  |
| Policies for regulation of e-cigarettes |  |  |
| **Other tobacco control policies and interventions (in place or planned)** | | |
| **2.9 Restrictions on retail availability or supply (eg, sales only in specialist stores)** | | |
| Specify policy or intervention  (add rows as necessary) |  |  |
| **2.10 Product information and modification (eg, menthol banned, compulsory listing of constituents)** | | |
| Specify policy or intervention  (add rows as necessary) |  |  |
| **2.11 Other policies or interventions (eg, nicotine reduction, tobacco-free generation, ‘sinking lid’ reduction in imports, pharmacy-only or prescription-only sales, removal of additives, smoker’s licence, ending sale of roll-your-own tobacco, comprehensive business district/city/village smoking bans, restructuring or increasing capacity of tobacco control sector and management)** | | |
| Specify policy or intervention – even if only proposed or under discussion  (add rows as necessary) |  |  |
| Restrict private importing of tobacco |  |  |
| Banning distance sales of tobacco products |  |  |
| Tobacco-free generation |  |  |

**APPENDIX TABLE 2. Country-specific smoking prevalence trends and endgame goals**

| **COUNTRY** | **ANNUAL RATE OF DECLINE IN CURRENT SMOKING PREVALENCE AMONG ADULTS 2005-2015 (WHO)*** | **DAILY ADULT TOBACCO SMOKING PREVALENCE IN 2018 **** | **YEAR ENDGAME GOAL WAS INTRODUCED** | **DAILY ADULT SMOKING PREVALENCE IN YEAR ENDGAME GOAL WAS INTRODUCED** | **ANNUAL DECLINE IN DAILY SMOKING (%) REQUIRED TO ACHIEVE 5% PREVALENCE BY THE ENDGAME TARGET YEAR** |
| --- | --- | --- | --- | --- | --- |
|  |  |  |  |  |  |
| **Canada** | -0.78% | 11%^2^  [16% current†]^2^ | 2018^1^ | 11%^2^  [16% current†]^2^ | 0.34% per year for 17 years  [0.64% per year for current smoking] |
| **Finland** | -0.58% | 12%^3,4^ | 2010^#^**^5^** | 18%^3^ | 0.58% per year for 12 years |
| **Ireland** | -0.84% | 17%^6^ | 2013^7^ | 21.5^^8^ | 1.71% per year for 7 years |
| **New Zealand** | -0.79% | 13%  (Note data are from 2017/18^)9^ | 2011^10^ | 16%  (Note data are from 2011/12)^9^ | 1.14% per year for 7 years |
| **Scotland** | -0.69% (UK) | 19% current ††^11^ | 2013^12,13^ | 21% current††^11^ | 0.88% per year for 16 years |
| **Sweden** | -0.76% | 7%^14^ | 2016^15^ | 9%^14^ | 0.29% per year for 7 years |

* Predictions from World Health Organization estimates extrapolating from available survey data using standardised methodologies.^16^ Of note these figures are in keeping with reported reductions in smoking rates over time in participating countries, please see the full ‘2018 INSPIRED Report’ at <https://aspireaotearoa.org.nz/our-research/current-research/inspired> for detail.

** As reported in population-based surveys, figures may not be fully comparable due to differences in sampling approaches and age-ranges of participants included in the surveys.

† Current smoking estimates are provided for Canada because in-country informants noted some doubts about validity of daily smoking prevalence estimates. The 16% estimate is preferred by Canada participants in this project, as it is considered more accurate than the daily smoking estimate. Similar figures were reported for over-15 year olds in the biennial Canada Tobacco, Alcohol and Drugs survey in 2017. ^17^

†† Current smoking estimates are provided for Scotland as no daily smoking prevalence data available.

^#^ The initial endgame goal was <2% prevalence of smoking and the target date was 2040. However, in 2016 the goal was updated to include all nicotine products, goal was set to less than 5%, and the target date was brought forward to 2030.

^##^ These targets were not met.

^ Data is from the smoking tracker survey which is conducted by the Health Service Executive. It is not directly comparable to the 2018 data which is from the Health Ireland Survey and used differing methodology.

^^ These targets were set in 2018. More detailed interim targets were also set in 2013.

**Table 2 references:**

1. Health Canada. Canada’s tobacco strategy (overview). Ottowa: Health Canada; 2018.
2. Statistics Canada. Health Fact Sheets: Smoking, 2018. Ottowa: Health Canada; 2019.
3. THL. Sotkanet.fi. 2018. <https://sotkanet.fi/sotkanet/fi/taulukko/?indicator=szY0CYo3BAA=&region=s07MBAA=&year=sy6rtDbS0zUEAA==&gender=m;f;t&abs=f&color=f&buildVersion=3.0-SNAPSHOT&buildTimestamp=201802280718> [Accessed 31 July 2023].
4. THL. Finsote-tutkimus 2017-2018. National FinSote survey. (In Finnish only); 2018.
5. Ministry of Social Affairs and Health. Roadmap to a tobacco-free Finland: action plan on tobacco control. Helsinki: Ministry of Social Affairs and Health; 2014.
6. Department of Health. Healthy Ireland Survey: Summary of Findings 2018, 2019.
7. Department of Public Health. Tobacco Free Ireland Report of the Tobacco Policy Review Group. Dublin: Department of Public Health; 2013.
8. Hickey P, Evans E. Smoking in Ireland 2013: synopsis of key patterns and trends. Dublin: Health Service Executive. 2013. <https://www.drugsandalcohol.ie/23200/> [Accessed 9 Nov 2023].
9. Ministry of Health. New Zealand Health Survey Annual Data Explorer 2018. <https://www.health.govt.nz/nz-health-statistics/surveys/new-zealand-health-survey#micro> [Accessed 9 Novermber 2023].
10. New Zealand Parliament. Government Response to the Report of the Māori Affairs Committee on its Inquiry into the tobacco industry in Aotearoa and the consequences of tobacco use for Māori (Final Response). Wellington: New Zealand (NZ) Parliament. 2011.
11. Scottish Government National Statistics. The Scottish Health Survey 2018 edition, volume 1: main report Edinburgh: The Scottish Government; 2020.
12. Scottish Government. Raising Scotland’s Tobacco-free Generation Our Tobacco-Control Action Plan 2018. Edinburgh: Scottish Government; 2018.
13. Scottish Government. Creating a tobacco-free generation: a tobacco control strategy for Scotland. Edinburgh: Scottish Government;
14. Ministry of Health and Social Affairs. A comprehensive strategy for alcohol, narcotics, doping and tobacco policy, 2016–2020. Stockholm: Ministry of Health and Social Affairs; 2016.
15. Public Health Agency of Sweden. National public health surveys, national and regional results. 2018. <http://fohm-app.folkhalsomyndigheten.se/Folkhalsodata/pxweb/sv/B_HLV/B_HLV__aLevvanor__aagLevvanortobak/?rxid=0715a352-2a64-44ee-9582-ce7709bbf53a> [Accessed 31 July 2023].
16. World Health Organization. WHO global report on trends in prevalence of tobacco smoking 2000-2025 (Second edition). Geneva: World Health Organization; 2018.
17. Statistics Canada. Canadian Tobacco, Alcohol and Drugs Survey (CTADS): summary of results for 2017. 2018. <https://www.canada.ca/en/health-canada/services/canadian-tobacco-alcohol-drugs-survey/2017-summary.html> [Accessed 31 July 2023].

**APPENDIX TABLE 3. Action plans/strategies to reach each the endgame goal in each of the participating countries**

| **COUNTRY** | **ACTION PLAN/STRATEGY** | **KEY TOBACCO CONTROL INTERVENTIONS FROM THE ACTION PLAN/STRATEGY**  **(interventions that were planned but not yet in place in 2018 are in blue)*** | **FOCUS ON ADDRESSING INEQUITIES** |
| --- | --- | --- | --- |
| **Canada** | Federal Tobacco Control Strategy, introduced in May 2018.^1^ | - Revamp smoking cessation support. - Explore options to further reduce the appeal and addictiveness of tobacco products through taxation, price interventions and regulation of nicotine content. - Introduce standardised packaging of tobacco products. - Development of a requirement for health warnings on individual cigarettes. - Development of federal regulations regarding e-cigarette advertising and promotion restrictions. - Development of strengthened federal regulations regarding tobacco industry reporting. - Explore a nicotine reduction strategy and applying costs of tobacco to society to the tobacco industry in Federal Control Strategy. - A new public education campaign targeting at-risk youth and young adults. | - Work with national and regional Indigenous groups to create specific plans for First Nations people. - Recognition of the need to create specific plans for First Nations people, incorporating their unique circumstances and seeking to continue and expand existing tobacco projects in Indigenous communities. |
| **Finland** | Roadmap towards a Tobacco-Free Finland, introduced in 2014.^2^ | - Reduction of the private importing of snus, and prohibiting use of tobacco at underage events. - Prohibit imports and sales of novel tobacco products. - Regular increases in taxes and prices of tobacco, with monitoring. Consideration of whether e-cigarettes should be taxed in the same way as other tobacco products. - Support for quitting, including free easily available cessation services and reimbursement of cessation medications as part of a health insurance scheme. | - Finland’s working group for the development of tobacco and nicotine policy proposed that cessation of tobacco and other nicotine products use among particular groups of heavy smokers should be enhanced in primary and specialised health services. - It has also been proposed that health care staff should do everything possible to reduce smoking and the use of other nicotine products among people recovering from mental health problems and many other specific groups. - Information campaigns, tailored to specific target groups. |
|  | Cross-sector Ministerial working group recommendations in 2018. Note that they are to be considered by the government, but do not yet represent government policy. | - Reduction of the private importing of snus and other tobacco products. - Bans extended to cover the smoking and heating of all products and any other use that releases aerosols deteriorating indoor air quality in all areas designated by the Tobacco Act. - Smoking bans extended to include outdoor places and areas that are mainly used by minors, such as playgrounds and EU beaches (apply to the use of oral tobacco). - Housing companies to ban smoking on balconies or indoors in facilities controlled by residents subject to majority vote. - Regular increases in taxes of tobacco and nicotine products. - Increase in minimum age to buy tobacco and nicotine products from 18 to 20 years. - Government monitoring systems to provide up-to-date information on the sale, consumption (including special groups), acquisition and marketing of nicotine products and imitations, cessation of nicotine product use, diseases and morbidity caused by their consumption, as well as the costs arising from these and their distribution over different population groups. - Plain tobacco products and packaging for tobacco, nicotine-containing liquids and refill containers and their retail packaging. - Ban on flavours for all tobacco and nicotine products. - Regulation of all nicotine products like tobacco products. - No public investments in tobacco or nicotine industry. - The responsible ministries to investigate and propose actions needed to prevent the environmental and health hazards posed by tobacco and nicotine products. - Reimbursing all prescription-only medicines used to treat tobacco and nicotine dependence under health insurance. |  |
| **Ireland** | Tobacco Free Ireland Action Plan, Introduced in 2015.^3^* | - Annual tax increases and reduction in the price differential on roll your own tobacco. - Standardised packaging of all tobacco products. - A ban on smoking in cars when children present. - Introduction of the legislative provisions of the EU Tobacco Products Directive. - Ring-fenced tobacco industry levy to fund health promotion and tobacco control activities - Increased social marketing campaigns. - Ban on menthol and flavours in tobacco products. - Retailer licensing. - Introduction of a ‘Track and Trace’ scheme to tackle illicit trade and ratification of WHO FCTC Protocol to Eliminate Illicit Trade in Tobacco Products. - Enhanced provision of smoking cessation support with targeting of key settings and priority groups. | - The 2013 tobacco control strategy states that smoking is the largest contributor to health inequalities between the richest and poorest sections of society, and that smoking is also a key factor in gender-based mortality differences. - The 2015 action plan includes targeting of smoking cessation support to people with lower socioeconomic status. - The goal is also endorsed in the 2016 Programme for Partnership, which states there is a high priority placed on reducing disparities, socioeconomic and among ethnic groups.^5^ |
| **Scotland** | Tobacco-Control Action Plan, introduced in June 2018.^4^** | - A focus on national campaigns to encourage quitting and protect children, in particular, from second-hand smoke. - Increasing service providers’ understanding and training on the links between smoking and mental health care. - Continuing to support ASH Scotland in promoting the Charter for a Tobacco-free Generation and encouraging Tobacco-free Schools. - There is also a commitment to explore options for new regulatory approaches in a variety of areas. - Very high priority on tackling inequalities in smoking, with a focus on deprivation and adolescents and young people aged 16-24 years. - Inclusion of up-to-date advice on e-cigarettes in the Health and Wellbeing strand of the school curriculum. - Ban on menthol-flavoured cigarettes, including roll your own tobacco. - Smokefree prisons with consistent through-care after release. - Smokefree hospital grounds – plan to make it an offence to smoke within 15 metres of hospital buildings. - Explore with local authorities and housing associations the idea of tobacco-free clauses in tenancy agreements and smokefree housing alternatives being offered in social housing. | - The 2013 tobacco control strategy included a commitment to carry out a Health Inequalities Impact Assessment to inform the strategy’s implementation. This has been completed and recommendations given to all stakeholders implementing the strategy.^6^ - The 2018 action plan had a strong focus on deprivation, with specified targets by deprivation group.^4^ |
| **Sweden** | Public Health Agency Action Plan with several specific measures to advance tobacco control, introduced in December 2018.^ | - Registration of tobacco retailers. - Tracking and tracing of tobacco products and ratification of the WHO FCTC Protocol to Eliminate Illicit Trade in Tobacco Products in July 2019. - Increased smokefree outdoor areas, including outdoor restaurants (from July 2019). - Promote cooperation in tobacco control (and alcohol and other drug) activities across Nordic countries. | - The 2016 national strategy for tobacco (along with alcohol and other drugs) states that equity (socioeconomic, gender, children) must be systematically taken into account in the strategy’s implementation.^7^ |

NB New Zealand does not have a formal Action Plan. However, upcoming plans include Smokefree cars legislation, formal regulatory framework for alternative nicotine delivery products, and further 10% above-inflation annual tobacco tax increases.

* Prior to this, the Tobacco Free Ireland Strategy was introduced in 2013.^8^

** Prior to this, the Tobacco-Control Action Plan was introduced in 2013.

^ Prior to this, the 2016 Alcohol Narcotics Doping and Tobacco Strategy^9^ contained broad statements regarding the need to reduce ‘illegal sales’ and marketing of tobacco products.

ASH: Action on Smoking and Health, EU: European Union, WHO FCTC: World Health Organization Framework Convention for Tobacco Control.

**Table 3 references:**

1. Health Canada. *Canada’s tobacco strategy (overview)*. Health Canada; 2018.
2. Ministry of Social Affairs and Health. *Roadmap to a tobacco-free Finland: action plan on tobacco control*. Ministry of Social Affairs and Health; 2014.
3. An Roinn Slainte Department of Health. *Tobacco Free Ireland Action Plan*. 2015. <https://health.gov.ie/blog/publications/tobacco-free-ireland-action-plan/> [Accessed 31 July 2023].
4. Scottish Government. *Raising Scotland’s Tobacco-free Generation Our Tobacco-Control Action Plan 2018*. Scottish Government; 2018.
5. First progress report: A programme for a partnership government 2016 - 2021. 2023. https://assets.gov.ie/4976/191218113714-2f566e1721fb4cf59199a1c67adda80b.pdf [Accessed 31 July 2023].
6. Scottish Government. *Creating a tobacco-free generation: a tobacco control strategy for Scotland*. Scottish Government; 2013.
7. Regeringens skrivelse 2015/16:86. En samlad strategi för alkohol-, narkotika-, dopnings- och tobakspolitiken 2016 – 2020 (in Swedish only). 2016.
8. Department of Public Health. *Tobacco Free Ireland Report of the Tobacco Policy Review Group*. 2013.
9. Ministry of Health and Social Affairs. *A comprehensive strategy for alcohol, narcotics, doping and tobacco policy, 2016–2020*. 2016.

**APPENDIX SUMMARY 1: Detail on Socioeconomic disparities in INSPIRED countries, up to 2018**

Examples of higher smoking prevalence being associated with lower income:

In Canada in 2017, smoking prevalence in households with the lowest income quintile was 21.7%. In houses in the highest quintile it was 11.9%.^1^

In Sweden, daily smoking prevalence for people on low incomes was 12% in 2018, compared with just 4% for those on high incomes.^2^

Examples of higher smoking prevalence associated with lower education levels:

In Finland in 2018, daily smoking was most common in the lowest educational group, with 17.6% reporting that they smoked daily basis. The corresponding value for people in the middle education group was 10.7% and for the higher education group it was 5.9%.^3^

In Sweden in 2018, daily smoking rates were 3% for women among the university-educated population, compared with 10% for high-school-educated and 14% for less than high-school educated.^2^

Examples of higher smoking prevalence associated with living in a more deprived area:

In Ireland in 2018, smoking rates were 26% for people living in more deprived areas, compared to 16% for people living in affluent areas.^4^

In New Zealand in 2017/2018, after adjusting for age, gender and ethnic differences, current smoking prevalence was three times higher for people living in the most socio-economically deprived compared to people living in the least deprived areas.^5^

In Scotland in 2018, 32% of adults living in the quintile with the most deprived areas were current smokers, compared with just 9% in the quintile with the least deprived areas.^6^

**References:**

1. Health Canada. *Health Fact Sheets: Smoking, 2017.* 2018.
2. Public Health Agency of Sweden. *National public health surveys, national and regional results.* 2018.
3. THL. Sotkanet.fi. <https://sotkanet.fi/sotkanet/fi/taulukko/?indicator=szY0CYo3BAA=&region=s07MBAA=&year=sy6rtDbS0zUEAA==&gender=m;f;t&abs=f&color=f&buildVersion=3.0-SNAPSHOT&buildTimestamp=201802280718> [Accessed 31 July 2023].
4. Department of Health. *Healthy Ireland Survey: Summary of Findings 2018*. 2019.
5. Ministry of Health. *New Zealand Health Survey Annual Data Explorer*. 2018.
6. Scottish Government National Statistics. *The Scottish Health Survey 2018 edition, volume 1: main report*. 2020.

**APPENDIX SUMMARY 2: The prevalence of alternative nicotine product use in INSPIRED Countries, up to 2018**

Canada

Current electronic cigarette (E-cigarette) use was 2.9% in 2017 (past 30-day use, ages 15 years and over). Daily use in 2017 was 0.8%.^1^

Finland

In 2018, about 1.4% of men and 0.3% of women used nicotine-containing e-cigarettes daily.^2^

Ireland

The Healthy Ireland Survey in 2018 showed 12% of the population had tried e-cigarettes at some point, and 4% were currently using them.^3^

New Zealand

In 2017/18 18.5% of adults had ever tried e-cigarettes, 4% were current users (used e-cigarettes at least once a month), and 3% were daily users.^4^

Scotland

In 2018, current e-cigarette use among adults was 7%, and 11% had previously used e-cigarettes.^5^

Sweden

‘Snus’ (a form of oral smokeless tobacco in pouches) is used, with 18% of men and 4% of women using snus daily in 2018.^6^

Further detail

Further detail, including prevalence figures by age and for people who smoke or quit smoking, is available in the 2018 INSPIRED Report:

<https://aspireaotearoa.org.nz/sites/default/files/2023-07/INSPIRED%202018%20FINAL%20REPORT_V1%20JUNE%202023.pdf>

**References:**

1. Statistics Canada. *Canadian Tobacco, Alcohol and Drugs Survey (CTADS): 2017 detailed tables Ottowa: Health Canada*. 2018.
2. THL. *Finsote-tutkimus 2017-2018. National FinSote survey.* (In Finnish only). 2018.
3. Department of Health. *Healthy Ireland Survey: Summary of Findings 2018*. 2019.
4. Ministry of Health. *Annual Data Explorer 2018/19: New Zealand Health Survey.* 2019.
5. Scottish Government National Statistics. *The Scottish Health Survey 2018 edition, volume 1: main report. Edinburgh: The Scottish Government.* 2020.
6. Public Health Agency of Sweden. *National public health surveys, national and regional results.* 2018.
